# “An illness of isolation, a disease of disconnection”: Depression and the erosion of we-experiences

**DOI:** 10.3389/fpsyg.2022.928186

**Published:** 2022-08-05

**Authors:** Lucy Osler

**Affiliations:** Center for Subjectivity Research, University of Copenhagen, Copenhagen, Denmark

**Keywords:** phenomenology, phenomenological psychopathology, depression, sociality, we-experiences, isolation, Ratcliffe

## Abstract

Depression is an affective disorder involving a significant change in an individual’s emotional and affective experiences. While the Diagnostic and Statistical Manual of Mental Disorders, Fifth Edition (DSM) mentions that social impairment may occur in depression, first-person reports of depression consistently name isolation from others as a key feature of depression. I present a phenomenological analysis of how certain interpersonal relations are experienced in depression. In particular, I consider whether depressed individuals are able to enter into “we-experiences” with other people. We-experiences are experiences had with two or more people as a *we* (rather than having an experience as an *I*), experiences that allow one to enter into robustly *shared* experiences with others. I claim that the ability to enter into we-experiences (both actual and habitual) is eroded in depression due to an overwhelming feeling of being different to and misunderstood by others. As such, I suggest that depression should be conceived of as fixing an individual in their first-person singular perspective, thus inhibiting their ability to experience in the first-person plural and to feel a sense of connectedness or togetherness with others as part of a *we*. By attending to on-going impacts of a diminished ability to enter into we-experiences, we can provide a situated and more nuanced account of the changes of interpersonal relations in depression that captures the progressive (rather than static) nature of the disorder. In turn, this analysis furthers our understanding of the emergence, frustration, and erosion of actual and habitual we-experiences.

## Introduction

Depression is an affective disorder involving a significant change in an individual’s emotional and affective experiences. While the Diagnostic and Statistical Manual of Mental Disorders, Fifth Edition (DSM) lists “depressive mood” as a core feature of depression, it does so “without fully considering what depressive mood is” (Ratcliffe, 2014, 5). Since the DSM definition of depression references various subjective experiences of depressed patients, it seems essential to ask what these experiences involve in order to understand the disorder. As David Karp puts it: “Research about a feeling disorder that does not get at people’s feelings seems, to put it kindly, incomplete” (2017, 67). Phenomenological research into depression has sought to reveal and analyze the experiential structure of the disorder (e.g., Fuchs, 2013a; Ratcliffe, 2014; Stephan et al., 2014; Maiese, 2018; Osler, 2021). Such an approach moves away from characterizing depression as something that someone merely has, to considering depression as something that individuals experience in their lived, situated interactions with the world and others.

First-person reports of depression consistently describe feeling isolated and alone as a key feature of depression. Karp goes as far to describe depression as being, at its core, “an illness of isolation, a disease of disconnection” (2017, 63). In this paper, I explore the feeling of isolation that characterizes many peoples’ experiences of depression by analyzing how interpersonal relations in depression are impacted. Specifically, I consider whether depressed individuals are able to enter into “we-experiences” with other people. We-experiences are a form of experience described in the phenomenology of sociality where two or more people experience something together as a *we*, rather than simply experiencing something as an *I* (e.g., Walther, 1923; Carr, 1987; Szanto and Moran, 2015; Zahavi, 2015). Such experiences are typically described as involving a *felt sense of togetherness* or *belonging* with the others involved. I suggest that the ability to enter into we-experiences is eroded in depression due to an overwhelming feeling of being different to and misunderstood by others. As a result, I suggest that depression involves a fixing of an individual in their first-person singular perspective, thus inhibiting their ability to experience in the first-person plural and to feel a sense of togetherness with others as part of a *we*.

In “Depression in the DSM,” I give a brief outline of how depression is defined in the DSM. In “Depression: a disease of disconnection,” I highlight how depressive experience is often characterized by a profound sense of isolation and connection from other people, drawing both on descriptions of depression found in David Karp’s book *Speaking of Sadness* (2017), as well as on the phenomenological research of Matthew Ratcliffe. I note that while there is much agreement that interpersonal relations are negatively impacted in depression, we are left with the question of which interpersonal relations are specifically impaired and how this accounts for the loneliness and disconnection experienced. To help narrow our search, in “What remains,” I draw attention to certain forms of being with other people that appear to remain intact in depression. In “We-experiences,” I set out what a we-experience is and what the conditions are for such an experience to arise. Using this analysis, I suggest in “Depression and the difficulty of self-alienation” that individuals with depression often report feeling that their experience is significantly different to others, as well as feeling that other people simply cannot understand them, and this inhibits the ability to enter into we-experiences and feel the sense of togetherness that such shared experiences entail. This allows us to point to a specific kind of interpersonal relation that is experienced as absent in depression. In the penultimate section, “The erosion of habitual we-experiences”, I argue that not only is the ability to enter in *new* we-experiences disrupted in depression but, over time, more *habitual* feelings of connectedness with others are also eroded. The erosion of both new and habitual we-experiences in depression renders the depressed individual a perennial outsider, able to observe, understand, and even interact with others but not able to richly share experiences and emotions with them, thus driving feelings of isolation and disconnection. I conclude with a discussion in the final section, “Social disconnection, depression, and other disorders”, of how my analysis might help us distinguish depression from other disorders, such as schizophrenia, which also involve experiences of social disconnectedness and isolation.

## Depression in the diagnostic and statistical manual of mental disorders

It is generally acknowledged that depression is an affective disorder. Descriptions of depression commonly refer to persistent feelings of isolation, loneliness, disconnection, anxiety, despondency, worthlessness, and hopelessness. The DSM states that at least one of the symptoms of depression is either:

i depressed mood most of the day, nearly every day, as indicated by either subjective report (e.g., feels sad, empty, hopeless) or observation made by others; or
ii markedly diminished interest or pleasure in all, or almost all, activities most of the day, nearly every day.

Alongside this, the DSM specifies that an individual must experience at least four of the following additional symptoms:

i significant weight loss or weight gain or decrease or increase in appetite nearly every day;
ii insomnia or hypersomnia;
iii restlessness or lethargy;
iv fatigue or loss of energy;
v feelings of worthlessness or excessive or inappropriate guilt;
vi diminished ability to think or concentrate, or indecisiveness; or
vii recurrent thoughts of death or suicide.

The DSM also mentions that depression can “cause significant distress or impairment in social, occupational, or other important areas of functioning” (American Psychiatric Association, 2013, 163).

Depression, then, is a disorder that is predominantly defined and diagnosed on the basis of subjective experience. Yet, although the DSM makes explicit reference to experiential disruptions in depression, its descriptions of them are only “cursory” (Ratcliffe, 2014, 5). This perfunctory reference to subjective experience in depression is reflected in much medical research. Even though depression is partly defined on the basis of lived experience, medical literature typically focuses on unearthing the biological dysfunction at the root of depression (Svenaeus, 2013; Ratcliffe, 2014). Now, I am not suggesting that depression has no biological basis. However, uncovering the experience of depression in the context of an individual’s situated interactions with the world and others often takes a backseat and this inhibits our understanding of what the disorder is and how best we should go about treating it.^1^

Over the last decade, there has been increased interest in how phenomenology can be used to analyze disturbances found in depression, including disturbances of temporality (e.g., Fuchs, 2013a; Ratcliffe, 2014; Maiese, 2018), personal identity (e.g., Svenaeus, 2013), bodily feelings (e.g., Fuchs and Schlimme, 2009; Fuchs, 2013a), and emotional experience (e.g., Slaby, 2014; Stephan et al., 2014). There has also been increased interest in phenomenological psychopathology in how depression involves impairments to one’s social experiences (e.g., Fuchs, 2005, Fuchs, 2013a; Ratcliffe, 2014; Ratcliffe and Stephan, 2014; Wehrle, 2019; Osler, 2021). I follow this trend here and present a phenomenological assessment of the erosion of certain interpersonal relations in depression. What my account offers is a specific consideration of how depressed individuals struggle to share experiences with others as a “we” and how this leaves individuals with a distinct loss of togetherness or connectedness with those around them. This, though, is not to suggest that social disconnectedness is the sole feature of depression, nor a suggestion that social disconnectedness of this kind is not a feature of other disorders. As such, my analysis is intended to be paired with other phenomenological descriptions of depressive experience to provide a full picture of what is distinctive about depression compared to other disorders. In this paper, I will not attempt to provide such a picture. However, I will return to the question of how this analysis might aid us in outlining what is distinctive about depression compared to other disorders, in particular schizophrenia, in the final section (“Social disconnection, depression, and other disorders”).

## Depression: A disease of disconnection

For the purposes of this discussion, I predominantly draw upon David Karp’s descriptions of the experience of depression in his book *Speaking of Sadness* (2017). While there are many memoirs dedicated to descriptions of depression, Karp’s draws not only from his personal experience but also from in-depth interviews with others who have struggled with depression. It should be emphasized, though, that depression is a complex disorder and the illustrations of depressive experience contained in Karp’s book should not be taken as exhaustive. Nevertheless, the descriptions of a sense of isolation, of feeling cut off from the world and other people, that I take as my focus, are reflective of descriptions found elsewhere (e.g., Plath, 1963; Wurtzel, 1994; Styron, 2010; Ratcliffe, 2014).

Depressive experience varies across individuals and has numerous different facets: from feeling isolated to profound lethargy to anxiety. Nevertheless, Karp notes that “the most insistent theme” (2017, 73) is the effect depression has on relations with other people. While interpersonal experience is by no means the only dimension of experience affected by depression, it is a significant and persistent feature of it. The following passages, taken from *Speaking of Sadness*, depict the experience of diminished interpersonal connection in depression:

During all this I felt deeply alone. Everyone else seemed to be moving through their days peacefully, laughing and having fun. I resented them because they were experiencing such an easy time of it; I felt utterly cut off from them emotionally. I was angry because there was no way they could understand what I was going through. Their very presence seemed to magnify my sense of isolation. (Karp, 2017, 59)
The irony…is that depressed persons greatly desire connection while they are simultaneously deprived of the ability to realize it. Much of depression’s pain arises out of the recognition that what might make one feel better – human connection – seems impossible in the midst of a paralyzing episode of depression. It is rather like dying from thirst while looking at a glass of water just beyond one’s reach. (Karp, 2017, 73)

As with all feelings and emotions, isolation is experienced in different degrees and hues. Some individuals feel obliged to withdraw from virtually all arenas of social life. Most people though, unless they become hospitalized, struggle through their daily obligations, sometimes heroically maintain a façade of “normalcy.” Others may continue to associate with friends and family while nevertheless feeling disengaged, uncomfortable, marginal, and profoundly alone. (Karp, 2017, 102)

The peculiarly painful mark of depression seems to lie in the desire to “connect” with other people while feeling robbed of the ability to do so. Indeed, Karp labels this the “paradox of depression” (2017, 91). What is more, Karp identifies a vicious feedback loop that often emerges in depression, where the inability to connect with other people causes such discomfort that depressed individuals ultimately withdraw from social life.

The breakdown or fracturing of social relations in depression has been highlighted in numerous phenomenological accounts (Fuchs, 2005, 2013a; Ratcliffe, 2014; Ratcliffe and Stephan, 2014; Wehrle, 2019; Osler, 2021). Perhaps most prominently, Matthew Ratcliffe, in his book *Experiences of Depression* (2014), describes how feelings of isolation, estrangement, distance from the world and other people are common experiences in depression: “The person is cut off from the world and, most importantly, from habitual forms of interaction with other people” (Ratcliffe, 2014, 31). He describes this profound sense of isolation as involving a loss of a “felt sense of connectedness to others” (Ratcliffe, 2014, 208). According to Ratcliffe, this amounts to a profound shift in how one experiences “being with” others and an erosion of “*certain kinds* of interpersonal relation” (Ratcliffe, 2014, 202, my emphasis). This leads us to ask *how* this loss of connectedness is experienced in depression and *what kinds* of interpersonal relations are impacted.

Ratcliffe claims that the loss of felt connectedness can be experienced in depression in two ways:

i where the depressed person retains a sense of what it is to connect with other people but feels that it is impossible to do so; and
ii a more profound alteration where the very sense of what it is to connect with others is lost altogether.

In (i), the possibility of connecting with someone is still anticipated but the ability to fulfill this connection is experienced as positively absent from the world. Interpersonal situations are experienced as *involving something missing* -- the absence itself is saliently felt.^2^ The alteration in (ii), in contrast, involves a loss of the very sense of what it is to connect with others – the possibility of connecting with others simply does not surface. This is a more extreme change in the structure of interpersonal experience.

Following Ratcliffe, I predominantly focus on the more commonly reported experience of feeling an absence of connection with others in depression (though I will touch upon (ii) at the end of section “The erosion of habitual we-experiences”). Generally speaking, then, a depressed individual:

…still anticipates experiencing the possibility of interpersonal connection when in the presence of certain others, and she “needs” this kind of connection, as her world is impoverished without it. However, whenever she encounters another person, the kind of relatedness she anticipates and/or needs is not experienced as possible. (Ratcliffe, 2014, 220)

Recently, a not dissimilar description has been offered by Tom Roberts and Joel Krueger of loneliness as an emotion of absence, as “an affective state in which certain social goods are regarded as out of reach for the subject of experience” (Roberts and Krueger, 2021, 185). The social disconnectedness that is described by depressed individuals often includes descriptions of an intense feeling of loneliness. Note, though, that Roberts and Krueger (2021) suggest that loneliness can be experienced as a temporary absence of social goods. Depression, though, seems to involve something more prolonged than a fleeting pang of loneliness – a more sustained experience of one’s being connected with others and the goods that such interpersonal relationships bring being eroded away. Importantly, one might be lonely without being depressed; either loneliness might be experienced relatively transient or one might experience loneliness as an experience of absent social possibilities without experiencing other markers of depression. Nevertheless, it seems that the kind of social disconnection that I am interested in in depression, may well be described as involving a persistent form of loneliness.

One distinction I am inclined to draw between the account here and that of Roberts and Krueger (2021) is that while I think the experience of being socially disconnected and isolated from others is an affective experience (Osler, 2021), I would hesitate to describe this experience of disconnection as an *emotion* of absence. Emotions are often thought to be relatively short-lived episodic experiences. Whereas the pervasive kind of social disconnectedness that individuals like Karp report, seems to be a more profound shift in how one experiences one’s social world and others. Following distinctions made in the phenomenology of emotions, we might be inclined to describe this disconnectedness in terms of an existential feeling or mood.

We are still left, however, with the question of what *kinds* of interpersonal relations are anticipated by the depressed individual that involve this sense of “connection” but are experienced as impossible to fulfill. While Ratcliffe states that there is an erosion of certain kinds of interpersonal relations, he does not explicitly explore which kinds of interpersonal possibilities are affected. There are many ways in which we find ourselves “with” other people and, as I argue in the following section, not all of these are eroded in depression. Indeed, for depression to retain its painful character, some of these experiences of being “with” other people must precisely remain intact.

## What remains

While depression is often characterized by feelings of isolation, disconnection, and loneliness, one is not thrown into an entirely solipsistic world. Others still feature in experience and depressed individuals do not seem to lose their ability to apprehend or understand others entirely. To help us home in on what kinds of interpersonal relation are disrupted in depression, I start by considering ways in which we find ourselves “with” others that typically remain intact in depressive experience.

### Physically being with others

Karp notes that depressed individuals often withdraw from social activities, spending lots of time on their own. However, he also emphasizes that the isolation of depression does not (usually) begin with physical seclusion. Indeed, the initial pain of isolation seems to be rooted in the feeling of isolation while being physically surrounded by other people. Take, for example, these two descriptions of isolation:

Oh, I was so alone. I played basketball. I was a member of a team. I had a roommate. But I was so alone. I had lots of friends but I was completely isolated. (Karp, 2017, 105)
Physically, I was not alone. As always Rose was present and listened with unflagging patience to my complaints. But I felt an immense and aching solitude. (Styron, 2010, 26)

Feeling isolated or disconnected does not neatly map onto physically being around other people or not; I can be at home on my own without feeling isolated or I can be in the middle of a busy party and feel completely alone (Roberts and Krueger, 2021; Tietjen and Furtak, 2021). Although the discomfort of feeling isolated while around others may prompt depressed individuals to avoid physically being with other people, the painful disconnection that is spoken of in depression does not appear to be grounded in being physically absent from others.

### Being with others in a shared world

Our sense of reality is intimately tied up with our experience of the world as a shared world. Part of what it is for me to experience the world, people, and objects as really there is that they are not just there for me but for other people as well; “I *experience* objects, events, and actions as public, not as private” in terms of only being there for me (Zahavi, 2003, 110). Being with other people is, at least in a weak sense of the phrase, implicated in our perception of objects and our experience of being in an intersubjectively shared world.

We might suppose that we could lose our sense of being in a shared world with others if we no longer experienced the world as available to other people. While not recognized by the APA, James Angelo suggests that astronauts can suffer “solipsism syndrome,” a syndrome described as “the state of mind in which a person begins to feel that everything around him or her is a dream and is not part of reality” (Angelo, 2003, 239).^3^ In such cases, individuals report experiencing the world as something that is derived from their own minds, rather than a public world that is available to all. Whether or not this is a bone fide syndrome, we can use this example to consider what it might be like to experience the world as only there for you. For in such a syndrome, any others would be experienced as also derived from your own mind, as not really there. It seems possible that such an experience could involve a total breakdown of interpersonal relations as people are no longer experienced as really existing anymore. This seems markedly different to the descriptions of social disconnectedness in depressive experience; while individuals report feeling isolated or cut off from the world and other people, they do not describe the world not being real or people as not really existing. It seems, then, that being part of a shared world in this basic sense, then, is not lost in depression.

### Sense of others as subjects

In our day-to-day lives, we do not experience the world littered by objects that we come to identify as other subjects but encounter the world full of other experiencing subjects (Stein, 1989). A loss of interpersonal connection, then, might arise where we to fail to recognize other humans as other subjects of experience. We can find such an experience in Capgras delusion. Those suffering from Capgras delusion experience other people not as other subjects but as “robots with human bodies” (Salviati et al., 2013, 139). Here, the patient no longer experiences being in a shared world with other experiencing subjects.^4^ We might also point to the lack of responsiveness and recognition of others as other subjects experienced in catatonia (Takaoka and Takata, 2007; Tandon et al., 2013).

While depressed individuals report feeling unable to connect with others, and even that they feel acutely separated from them, this does not amount to an experience of others as being object-like automatons or not there at all. Indeed, not only does the depressed person still experience other people as experiencing subjects but this seems to be an essential part of the experience of feeling estranged from or cut off from other people. If the depressed person no longer experienced people at all, it does not seem to make sense to say that they feel cut off from people as they would be no people to be cut off from. Moreover, depressed individuals often seem sensitive to the kinds of experiences others are having and feel their own situation to be in stark contrast. Think of Karp’s description of how he experienced other people as happy, as carefree, as being able to go about their business in an easy manner. The pain of his own unhappy experience was intensified by his awareness of other people’s experience. The loss of connectedness, then, does not seem to arise from an inability to either recognize others as experiencing subjects or from a total loss of the ability to apprehend how others are feeling or what they are doing.

## We-experiences

The previous section helps show that not all kinds of interpersonal experiences of “being with” other people are diminished in depression. How, then, might we account for the profound sense of disconnection and loss of togetherness reported by depressed individuals? I now want to turn to a specific kind of interpersonal experience that has recently received a lot of attention in the phenomenology of sociality – shared or we-experiences. The term we-experience is used to denote experiences that we have together *with* others, experiences that we *share*. This strikes me as a promising line of inquiry as we-experiences are typically described as involving a felt sense of togetherness with other people, an affective experience of being experientially unified with others (e.g., Walther, 1923; Szanto, 2016; León et al., 2019).

David Carr, in his 1987 paper “Cogitamus ergo sumus,” notes that colloquially there are many ways in which we use the word “we.” The phrase “we saw the Eiffel Tower” might simply mean that I have seen the Eiffel Tower on one occasion and that you have also seen the Eiffel Tower on another occasion. In this instance, we have both seen the Eiffel Tower but our sightings took place at different times. Carr suggests that here one could quite happily replace the “we” in this expression with “you and I.” However, Carr argues that this substitution is not appropriate if the phrase “we saw the Eiffel Tower” is meant to capture that we saw the Eiffel Tower *together*. In this second case, there is something lost in the substitution. What Carr is highlighting is that there is a phenomenological difference in experiencing something on one’s own compared to experiencing something together with someone else. When referring to experiences we had together, the word “we” is not just shorthand for “you and I” but designates a particular *kind* of experience that we had with someone else, where the experience of seeing the Eiffel Tower was not just *my* experience but *our* experience.

The term “we-experience” designates a special kind of experience that is “no longer simply experienced by me as *mine*, but as *ours*” (Zahavi, 2015, 90). Discussions of robustly shared we-experiences have their roots in classical phenomenological work, such as Edmund Husserl, Max Scheler, and Gerda Walther. Here, I will focus on the more contemporary work of Dan Zahavi, who has written extensively on this topic. My reason for doing so is primarily practical, as Zahavi has devoted considerable time and pages to clarifying what early phenomenologists meant by this term and spelling out the requisite conditions for a we-experience to emerge. Zahavi (2015, 2019) argues that experiencing something with another together as a “we” involves: (i) reciprocal other awareness and (ii) integration (also see León et al., 2019). Let us look at these two conditions in turn.

### Reciprocal other awareness

In order for a we-experience to arise, the first thing we need is at least one other person.^5^ If we are to see the Eiffel Tower *together*, I must be aware that you are there. Importantly, though, it is not enough for me to simply be aware that you are also present. I could be standing behind you and aware that we are both looking at the Eiffel Tower but this would not be sufficient to characterize our experience as looking at the Eiffel Tower *together* as you have no idea that I am there at all. You must *also* be aware of me looking at the Eiffel Tower. Crucially, though, what we need is not just parallel awareness of one another – for I might be aware that you are there, you might be aware that I am there, but neither of us are aware that we are attending to one another (e.g., if we keep glancing at each other but without noticing the other’s glances). What is required is a *reciprocal* awareness, where both you and I are mutually aware of being attended to by the other (Zahavi, 2015, 2019).

While reciprocal other awareness is required for a we-experience to arise, it is not sufficient. For instance, I could be eating lunch in a café at the same time as you and we might be reciprocally aware of each other. However, at this stage it is not appropriate to say that we are eating lunch together. What Zahavi claims is missing here is the *integration* of our experiences in an appropriate way that transforms our experience from the first-person singular to the first-person plural (Zahavi, 2015).

### Integration

While the notion of reciprocal other awareness is relatively simple, what does it mean for our experiences to become integrated? Broadly speaking, the word integration is used to capture how two people’s experiences come together in a way that transforms the experience from one that is had as an “I” to an experience that is had together as a “we.” The starting point for this integration of experiences is that it must make a difference that we are experiencing something together rather than alone. Zahavi describes this in terms of the individuals involved feeling that the “structure and quality” (2015, 90) of their experience is impacted by the other’s experience. When two strangers are looking at the Eiffel Tower, they might have reciprocal awareness of one another but just happen to be gazing at the Eiffel Tower at the same time. This would be a case of coinciding or parallel experiences. Contrast this with two friends who have come to gaze upon the structure. In looking at the Eiffel Tower, they direct one another to different aspects of the tower, enriching each other’s appreciation of it, enjoying the experience more because it is something that they are discovering together rather than apart. In this second case, part of what it is for the friends’ experiences to become integrated with one another is that their experience of seeing the Eiffel Tower is intertwined with and interdependent on one another’s experience. In other words, it *matters* that the other person is involved.

However, intertwinement and interdependence of experience will not suffice. If I trip over outside and someone laughs at me, my embarrassment is intensified by the other’s mirth. We could not have had this experience apart – my embarrassment is bound up with their seeing me and their delight is bound up with watching me fall. Yet, this does not amount to a we-experience. The integration we are looking for is *a special kind of integration*, one that involves what Gerda Walther (1923) calls an “inner bond” or “feeling of togetherness.”

What, then, does this special kind of integration involve? Note that this integration is not meant as some kind of fusion of experience into some group mind or group consciousness – the participants are not coming together as *one undifferentiated subject*. Zahavi emphasizes that “[a] we, a first-person *plural*, is not an enlarged I” (Zahavi, 2019, 5). Experiencing something together as a *we* involves a particular kind of relation *between* the participants and a “relation between” implies a plurality of participants. Nevertheless, “the difference between self and other cannot remain *too salient*, since this will prevent the required unity and integration from actually happening” (Zahavi, 2019, 5, my emphasis). For a we-experience to occur, each participant’s experience is transformed in a way that emphasizes their similarities while downplaying the differences (Zahavi, 2015, 2019; León et al., 2019), so they each come to experience themselves as “one of us” who is having the experience.

Zahavi suggests that you cannot enter into a we-experience if you are rooted in your first-person singular perspective; what is characteristic of the first-person singular perspective is that one’s experience is given exclusively as *mine*, whereas in a we-experience, the experience is given to the participants as *ours.* This transformation occurs through “self-alienation.” Self-alienation has rather negative connotations, however, it is used here to capture a process that involves appreciating and adopting another’s perspective on oneself (Zahavi, 2019, 6). As I understand it, the alienation that occurs is a *distancing* from your own I-perspective through an incorporation of another’s perspective on you. This is rather a tricky concept to articulate, however, I suggest it is one that we are all familiar with.

Let me illustrate this with an example: I am sitting doing a jigsaw puzzle. I am struggling to find the right pieces, have a pain in my back from sitting at the table for a long time, and am starting to lose interest in the whole thing. My experience is suffused by a variety of experiences *from my perspective*. Then you come along and start putting in pieces of the puzzle too. I understand that your perspective on me is “you are doing a jigsaw puzzle” and I have the same perspective on you. Under your gaze, I appreciate your perspective of me and I can adopt and identify with your perspective on me as doing a jigsaw puzzle in a way which downplays the frustration and discomfort that I experienced before you came in.

What Zahavi claims is that through self-alienation I not only achieve some distance from my I-perspective but can also come to *feel myself as like you*. If I experience myself through your eyes as “doing the puzzle” and experience you as “doing the puzzle,” I can come to experience myself as *one of us* doing the puzzle. By experiencing myself through your eyes, my own I-perspective is “downplayed.” Rather than experiencing the situation as “I am doing this” and “You are doing that,” the *I* recedes into the background in favor of a sense of mutual, shared experience; those elements of the experience that only feature for me (for instance my back pain or frustration) are less prominent than the elements that we are experiencing together (the doing of the jigsaw puzzle). In this way, the similarities between myself and the other are accentuated and the differences minimized, giving rise to an experience of doing the puzzle *together* as a “we.” Importantly for our purposes, a characteristic feature of experiencing something as a “we” is that we not only have a similar experience to another person, we *feel* a sense of togetherness or connectedness with them.

At first glance, the transformation from an I-perspective into a we-perspective looks rather laborious. Zahavi requires us to first appreciate the other’s perspective on oneself, identify with it, and incorporate it into how one sees oneself thus distancing oneself from one’s I-perspective and shifting to a we-perspective. However, he argues that this transformation typically does not occur through onerous cognitive reflection but arises pre-reflectively.

### Failures of integration

We can enrich our understanding of how we-experiences arise and the notion of self-alienation by considering how the requisite integration might fail to come about. Returning to our puzzle example, imagine that my back pain happens to be very severe. This pain is only experienced by me, it is not something common between the two of us.^6^ Where this difference in my experience and your experience is so prominent, it seems unlikely that the similarities between us will be accentuated enough for us to come together in a shared first-person plural perspective; my own exclusive experience is too pronounced to fade into the background in the requisite manner. I think it likely that this kind of pain might jeopardize self-alienation from the I-perspective and hinder identification with the other as a ‘‘we.’’ This illustrates how one might remain ‘‘rooted’’ in one’s first-person singular perspective.^7^

I also think that my adoption of your perspective on me will only take place if I take you to understand me with a degree of *accuracy*. Say I am sitting at the table sorting through jigsaw pieces looking for particular shades of green I like because I want to repaint my apartment. You come along and I am aware that you think I am just sorting the jigsaw pieces into colors in order to complete the jigsaw. If your perspective on me is that I am “doing the puzzle” when I am actually examining the pieces because I like the color of them, I am not likely to adopt your perspective on me. It seems difficult to see how we could come to share an experience of doing the puzzle together if I think you have mistaken what I am doing or experiencing; there seems to be little ground here for anything to be shared. Indeed, that you seem to have misapprehended what I am doing can make me feel a sharp contrast between myself and you. As such, I take it as necessary that I must feel *understood* by you if I am to adopt this self-alienating perspective on myself and come to identify as “one of us.”

## Depression and the difficulty of self-alienation

Having outlined the conditions for the emergence of a we-experience, let’s explore how entering into a we-experience might be inhibited in depression. As detailed above, entering into a we-experience with others involves (i) reciprocal other awareness and (2) an integration of the participants’ experiences into a “we.” As noted above, depressed individuals do still experience others as experiencing subjects in the world. It does not seem to be the case, then, that depressed individuals are incapable of having reciprocal other awareness with others. Being depressed does not stop me being aware of your presence and aware that you are also aware of me.

What about the second condition: integration? As discussed above, part of what is required for one’s own experience to become integrated with another to form a “we” is the feeling that one’s own experience is intertwined with and independent on the other’s experience, that the participants are mutually affecting one another. Some claim that in depression, individuals no longer feel themselves affectively moved by the emotions or bodily actions of actions, resulting in a loss of “interaffectivity” between subjects (e.g., Fuchs, 2005; Varga and Krueger, 2013). Think, though, of the descriptions that Karp gives of the pain of being in the presence of others when depressed, how seeing others smoothly engage with the world makes one’s feeling of isolation even more acute. In a broad sense, then, depressed individuals do still seem to be affected by others’ experiences, even if it is in terms of feeling more profoundly disconnected and distant from them.

However, a special integration is required for a we-experience. As Zahavi puts it, for two (or more) people to become integrated into a “sense of us,” individuals need to be able to downplay their experiential differences in favor of their similarities. I suggest that in depression the difference between oneself and others is experienced as *too salient* for the requisite integration to take place. In the following, I explore two features of depressive experience that might disrupt the kind of self-alienation required for we-experiences: (i) the prominence of one’s own exclusive “I” experiences and (ii) a profound feeling of being misunderstood by others.

### The prominence of “I” experiences

As discussed in the jigsaw puzzle example, if experiences that are *exclusively mine* are too prominent, this might hinder the ability to distance oneself from one’s first-person singular perspective. I used severe back pain as an example of an experience that is *mine* (and not yours) which might be experienced as too persistent and prominent to downplay in favor of the experience that *we* are doing a jigsaw *together*. Even when performing something that from the outside might look like a we-experience, I might be focused upon my back pain and thus “fixed” in my I-perspective. When this occurs, the similarity between our experiences that arises in the context of doing a common activity is not sufficiently strong to outweigh the felt difference between what *I* am experiencing and what *you* are experiencing.

Many felt dimensions of depressive experience might give rise to prominent and persistent experiences that make it hard to feel the similarity between oneself and others. For instance, feelings of tiredness, lethargy, anxiety might all be experiences that are hard to distance oneself from. These prevalent, and importantly unshared, experiences of *mine* might prove too difficult to downplay. Even where one is involved in some kind of collective activity, one’s I-perspective might continue to be *too salient* for the integration and transformation of one’s experience of doing or feeling something together as a “we.” This, I think, nicely captures Karp’s descriptions of how a depressed individual might continue playing basketball with friends as part of a team, an activity that looks like it might provide fruitful ground for the emergence of a we-experience, while no longer feeling a sense of togetherness with the other players. The felt absence that is experienced is the missing feeling of togetherness or connectedness that arises when we do or feel things with others as a “we.” This absence might be felt particularly strongly when individuals are taking part in a common activity, an occasion where one might expect a sense of togetherness to manifest, and yet this connectedness fails to come about, leaving the depressed individual with a distinct sense that something is off, that something anticipated is missing. This feeling of absence can, in turn, fuel feelings of frustration, anger, even resentment at the ease with which others seem to connect with one another. This piles on more affective experiences that mark the depressed person as different to others, experiences that further fix them in their own I-perspective. Thus, we can see how a vicious cycle can emerge as one reacts with disappointment and anger to one’s experienced lack of connectedness. This captures the very paradox of depression that Karp describes – the real desire and even need to connect with others, while feeling oneself incapable of achieving this connection.

In a similar vein, there are a number of studies that suggest that depressed individuals are prone to ruminate on their experiences (e.g., Takano and Tanno, 2009; Krieger et al., 2013). Rumination involves repetitive and reoccurring thoughts focused on one’s own experiences (and symptoms). As Krieger et al. (2013, 502) note, “brooding (referring to self-critical moody pondering) has been shown to be associated with higher levels of depression.” Such rumination seems to further embed someone in their first-person singular perspective, making it hard to appreciate and adopt the other’s perspective oneself and to distance oneself from the “I” in a way that allows for a shared experience with another as a “we” to arise. Rumination also seems to debar self-alienation. Moreover, it seems plausible that if a depressed individual feels a lack of connection with others, they might critically brood upon this change in their interpersonal experiences. This might lead to a viscous circle where depressed individuals increasingly reflect on their sense of isolation while around other people, thus accentuating feelings of being different from others and inhibiting their ability to feel togetherness with others as part of a “we.”

### Feeling misunderstood

Feeling profoundly misunderstood by others is another common symptom of depression. As one of the reports from Karp (2017, 59) puts it: “I felt utterly cut off from [other people] emotionally. I was angry because there was no way they could understand what I was going through”. This feeling of being misunderstood does not simply refer to instances where someone mistakes or misinterprets what a depressed individual is doing or experiencing. Rather, it is a profound sense that *no-one* is able to understand their depressed experience, that others *cannot* understand their experience of the world as drained of connection, significance, hope or energy. The feeling of not being understood that marks depression is often experienced as inevitable and irreversible.

As mentioned above, feeling misunderstood stymies we-experiences. If a depressed individual takes their own experience to be unlike the experiences of others, there seems little ground for experiencing oneself as “like others.” Moreover, if a depressed individual does not think that other people can ever understand them, they are unlikely to adopt and incorporate other peoples’ perspectives of them. This deep sense of feeling of being misunderstood, then, seems to prevent the kind of identification with others that is involved in a we-experience. Again, the depressed individual experiences themselves as *too different* to others for a we-experience to emerge, leaving the depressed individual feeling like an outsider, cut off from the rich feeling of sharing experiences and emotions with others.

## The erosion of habitual we-experiences

Even if persuaded by the argument that the ability to enter into we-experiences is inhibited in depression, some might wonder how helpful this is for more broadly understanding interpersonal experience in depression. As Zahavi (2019) himself remarks, we-experiences are not ubiquitous experiences. Indeed, some even suggest that we-experiences might be quite peculiar or even rare occurrences (Szanto, 2018) (though I am inclined to think that this is not the case). It might seem to some that my analysis only points to a small subspecies of interpersonal relation that is eroded in depression. How, then, can this help account for the profound and pervasive sense of disconnection from others that depression involves? In this section, I explore how difficulties with self-alienation and identification might not only prevent depressed individuals from experiencing new we-experiences with others but might have a detrimental effect on more habitual feelings of togetherness. Understanding this more habitual sense of togetherness captures how our intersubjective worlds often are experienced as marked with a connectedness with others, if when we are not engaged in an explicit we-experience, even when we are alone.

### Habitual we-experiences

Gerda Walter notes that the feeling of togetherness experienced in what she calls “actual we-experiences” can often “dissolve quickly” (1923, 48). However, she does not conclude from this that we-experiences only ever give rise to transitory feelings of connectedness and belonging. Rather, she suggests that in certain relationships, the feeling of togetherness of a we-experience does not simply disappear but, over time, can be *sedimented*. When this occurs, we come to experience a background, habitual sense of togetherness with those individuals without the need for a full we-experience to occur.

Take our friends who were looking at the Eiffel Tower together. Imagine these friends continue to travel together, exploring the sights of France and beyond. Over time, their experience of being “one of us” becomes second nature:

Just as certain intense and lively emotions (e.g., love) can sediment themselves and transform into more habitual states of mind, so can a similar sedimentation take place in the case of unification. To see this, think of the difference between the feelings of unification or togetherness characterizing a friendship – fervent, lively and constantly reinforced at the beginning, they eventually become sedimented background-feelings. Unification, in this case, is first explicit, but becomes habitual over time. (Zahavi and Salice, 2017, 520)

With some people we do not need to continually ‘‘rediscover’’ our sense of togetherness with them through the performance of a we-experience. That sense of togetherness becomes a background feeling that pervades our relationship.^8^ According to Walther, this background sense of togetherness is more common than actual we-experiences (1923, 46).^9^

The idea that we experience a habitual sense of *we*-ness in some of our interpersonal relations becomes more apparent if we consider what it is like if this habit is disrupted:

We know that a habit of togetherness forms because once it is ruptured, either by conflict or death, one experiences a profound loss or undeniable change…The death of a lover, for example, results in an acute awareness of how one is used to existing in and relating to the world. (Calcagno, 2012, 100).

With the loss of a lover, the background sense of connectedness with them that we are used to feeling is revealed through its absence. We can also think of less extreme examples where this occurs, for instance, when we drift away from certain friends, or in cases when someone is excluded from a friendship group. When we take into account these habitualized experiences of togetherness with other people, we capture a deeper, more implicit, sense of belonging that we often experience with friends and family – a “sense of us” that is more pervasive, and less demanding, than the more explicit we-experiences described above.

### The erosion of habitual togetherness in depression

Why is this discussion of habitual togetherness relevant to us? I think it points to a broader sense of connectedness that is also vulnerable to corrosion in depression. While the identification with another as a “we” might become second-nature with certain people, it is not immune to conflict. While Walther does not expound upon this, habits are neither determinate nor fixed. While I might have a habit of running down my stairs every morning, if I sprain my ankle, I am not able to act upon this habit. If my ankle is painful for a long period of time, my habit of running down the stairs may even disappear and even after my sprain has recovered I may continue to walk down the stairs rather than run down them. This habit may also be disrupted if my environment changes and I move to a ground-floor flat.

To be sustained, habits need to be enacted, otherwise they might change, disappear, or become disrupted (Maiese, 2016; Candiotto and Dreon, 2021). A habitual feeling of togetherness, an example of what Candiotto and Dreon (2021) describe as a “habit of feeling,” can also be dislodged. Think of how our traveling couple might get into an argument one day. Their habitual identification with one another as a “we” might be replaced by a feeling of being at odds with the other. Their background feeling of togetherness is not, then, impervious; it can come into conflict with other experiences. It might be that the couple’s habitual sense of *we*-ness is quite robust and perhaps the argument ruptures their sense of togetherness in that moment but their deeper sense of being “one of us” is not permanently eroded. As Lohmar (2014, 52) remarks, habits can be “sluggish” to change. Nevertheless, if arguing becomes common between them, a “crisis of habit” (Candiotto and Dreon, 2021) might occur where there is a conflict between the tense situation between the pair and the hitherto experienced habitual sense of togetherness. If this crisis continues, the sedimented sense of togetherness might eventually evaporate and instead be replaced by a sense of disconnection from each another.^10^

Just as Zahavi anticipates that where differences are felt too saliently this will jeopardize a felt sense of togetherness in an actual we-experience, I suggest that differences felt too saliently will, over time, also disrupt the feeling of togetherness in habitual we-experiences. If someone consistently experiences their own I-experiences as hyper-salient, unique to themselves, and even not understandable by others, this can work to unseat these more implicit feelings of togetherness. Where someone with depression feels that their experience of the world is so alien to their friends, partner, or family, seeing the other no longer awakens the habitual sense of connectedness, they feel their own experience as in contrast to the other, as inaccessible and not shareable between them. Just like how an argument can temporarily come into conflict with our usual sense of togetherness with a loved one, this might not immediately dissolve that sense of togetherness. However, if this feeling of being different to the other continues and the habitual sense of togetherness is no longer sustained or revitalized, it will weaken and erode. We might also suppose that as depressed individuals feel increasingly disconnected from those around them, they get caught in a viscous feedback loop, where they feel increasingly like their experiences are markedly different to other people and that there is no chance that others will understand what their experiences are like.

The loss of these background feelings of togetherness are likely to feel especially painful as they often involve a disruption of personal, long-term interpersonal relations. Not only is there a felt absence of a usually present togetherness, the absence itself is experienced as something unfamiliar. This might help us make sense of Ratcliffe’s claim that depressed individuals *expect* there to be a sense of connection with others but experience it as impossible to fulfill. It is not just that we go around expecting to strike up new we-experiences with everyone we meet but that many of our day-to-day interpersonal relationships are *usually* characterized by a sense of connectedness resulting from the sedimentation of actual we-experiences. When our habitual feelings of togetherness are disrupted, it can change how we find ourselves in the world, rendering it strange and unfamiliar.

By accounting for how habitual experiences of we-ness, or togetherness, are eroded in depression, I think we get closer to understanding how being rooted in the first-person perspective might give rise to a profound sense of disconnected from other people. If a felt sense of being different to and misunderstood by others comes into conflict with sedimented feelings of togetherness, we can appreciate that it is not only *new* interpersonal relations that are affected but also our *already established* interpersonal relationships.

Interestingly, the framework of habit also allows us to account for how different interpersonal relations are differently affected in depression. The time I spend with my partner as a couple, as well as the quality of our connection and companionship, is likely to sediment a deeper sense of togetherness between us than I experience with my work colleague. Thus, we might predict that in an episode of depression it will take longer for the sense of togetherness with my partner to completely erode than in the case of my work colleague. We might also predict that the disruption of my sense of being *one of us* with my partner will be more unfamiliar, more strange, more painful. This, then, helps capture how interpersonal relations breakdown at different rates and intensities in depression.

Adding habitual feelings of togetherness into the picture helps capture the “downward spiral” of depression (Karp, 2017, 91). A person does not usually wake up one day in the throes of major depression. Rather, depression is something that progresses, that, unless treated, gets worse. The idea that there are gradations of sedimented we-experiences might account for how the feeling of connectedness to others slowly seeps out of the world (rather than evaporating all at once). Moreover, it introduces the idea that over time, as habitual feelings of we-ness erode and new we-experiences fail to arise, we may come to experience a habitual sense of *dis*connection.

Where a habitual we-experience is unsettled by an inability to distance oneself from one’s first-person singular perspective, this does not necessarily immediately erode that habit. So, when we see that person again, we might still expect to feel a sense of connection with them that is then (painfully) disappointed. This fits Ratcliffe’s description of the depressed person who retains a sense of what it is to connect with other people but feels that it is impossible to do so. However, over time, as the habit of feeling a sense of togetherness with others is eroded it might be replaced with a new habitual sense of *dis*connection. This formation of a new habit of disconnection might help us understand how a depressed individual transitions from retaining a sense of what it is to connect with others to the more profound alteration where the very sense of what it is to connect with others is lost.

In the later stages of major depression, the sense of disconnection becomes the new “normal” and not only does the ability to enter in we-experience disappear, but the very possibility of such relationships evaporates, leaving the depressed individual further cut adrift from other people. What might start off as occasional episodes of failing to connect with others can sediment into a more pervasive sense of the acute absence of connection and finally cement into a hopeless sense of disconnection. As depression progresses, the very sense of what is possible alters. Revisiting what I said above, one way to conceive of this progression is to think of depression as involving an alteration in mood or existential feeling, a very shift in the way the world affectively unfolds around us and presents us with the possible (Ratcliffe, 2014). When depression is at its most serious, the very intersubjective world upon which the possibility of we-experience rests, can itself be eroded leading to a deeper and more profound rupture of intersubjectivity. Taking both actual and habitual we-experiences into account, therefore, not only helps us understand what kinds of interpersonal relations might be disrupted in depression that results in isolation, but also helps us construct a picture about how depression progresses. This, I think, not only enriches our understanding of social impairment in depression but also reflects depression’s progressive, rather than a static, character.

## Social disconnection, depression, and other disorders

As the reviewers of this paper both pointed out, social disconnection and impaired intersubjectivity are also reported in disorders other than depression, such as autism (Krueger and Maiese, 2018; Boldsen, in press), schizophrenia (Fuchs, 2005; Sass and Pienkos, 2013; Van Duppen, 2017; Salice and Henriksen, 2021), and (delusional) melancholia (Fuchs, 2005). In acknowledging this, though, we do not want the boundaries between these disorders to collapse. How, then, might we recognize that disrupted intersubjectivity is a feature of more than one disorder, without losing the experiential differences between them? My answer to this is threefold:

i while allowing that impaired ability to enter into and sustain we-experiences might occur in other disorders, what impairs this ability might be different;
ii different forms of interpersonal relationship might be impacted in different disorders, which impacts how social disconnectedness and disrupted intersubjectivity are experienced; and
iii there may be other characteristic features of a disorder, such as temporal experiences, bodily experiences, experiences of agency and autonomy, that differentiate it from depression despite involving a similar experience of social disconnectedness. To illustrate these points, I will consider how depression differs from schizophrenia (though I take it that this tripartite framework is applicable to other disorders as well).

First, there are reasons to suppose that schizophrenic individuals might also struggle to enter into we-experiences with others (Van Duppen, 2017; Salice and Henriksen, 2021) and, thus, my analysis of how we-experiences can be disrupted might also be helpful for exploring social disconnection in schizophrenia as well as depression. However, it is interesting to note that we-experiences can fail to arise for reasons other than those I have detailed above (i.e., the prominence of “I” experiences, rumination, and feelings of being misunderstood). According to practitioners such as Minkowski (1970) and Fuchs (2013b), schizophrenic individuals often experience temporal fragmentation that results in “reduced attention spans, disturbances in planning, initiation, sequencing and synchronization of speech as well as in the performance of other activities” (Fuchs, 2013b, 88). This temporal fragmentation can also give rise to what Fuchs describes as damaged basic self-coherence which disrupts the flow of temporal experience and threatens a sense of continuous selfhood and mental life. Fuchs suggests that a consequence of this is that schizophrenic patients experience “difficulties in recognizing faces and in interpreting facial expressions or gestures” (2013b, 92). This temporal rupturing seems likely to hinder the emergence of we-experiences in various ways, including inhibiting the individual’s ability to empathetically perceive what the other is doing and experiencing (an essential component for we-experiences as detailed above), as well as inhibiting the ease with which an individual might engage in shared activities and joint attention – activities that are fertile grounds for the emergence of a “we.” Importantly for our purposes, while an inability to enter into we-experiences might be a feature of schizophrenic experience that is shared with depression, what gives rise to the disruption appears to have certain differences to the case of depression. This, then, might account for both an overlap between the disorders, while maintaining their distinctive experiential features.

Second, there might be other intersubjective relations hindered in schizophrenia that remain intact in depression. As mentioned above, there is evidence that schizophrenic individuals often struggle to understand the expressions and gestures of others. This diminished ability to understand others is likely to give rise to social disconnectedness, leaving the schizophrenic individual in a world of uncertainty about others’ experiential lives. This, then, might be an additional dimension of intersubjective disruption in schizophrenia that contributes to a broader sense of isolation but one that is not shared with depression. Adopting a fine-grained analysis of exactly *how* social relations are affected in various disorders will help us map out differences, as well as similarities, between such disorders.

Third, while I have argued that social disconnectedness is a common (and often central) feature of depressive experience, this is not to suggest that this is the only feature of depression. Distinctions between depressive experience and schizophrenic experience, then, can also be found by attending to additional features of these disorders – such as temporal experience, bodily experience, agency and autonomy, and other affective experiences. For example, the occurrence of thought insertion and disturbed for-me-ness (as detailed by Henriksen et al. (2019)), are considered by many to be characteristic features of schizophrenia but not of depression. As such, we can also account for experiential differences between disorders by looking beyond social dimensions to other distinctive features.

It should also be noted that experiences of social disconnectedness can flow from a disorder due to stigmatization. In certain instances, we might not want to suggest that feelings of isolation, disconnection, and loneliness are necessarily a core characteristic of a disorder while also recognizing how the stigmatization of various “mental health” disorders can leave individuals at risk of social exclusion and social stereotyping (Osler and Krueger, 2021).

All of this is to say that experiences of social disconnectedness, isolation, and eroded or disrupted intersubjective relations can come in many flavors and the experiential dimension of disorders are typically complex and textured. As such, I think that my analysis of the erosion of we-experiences may help us identify similar disruptions in other disorders, without the risk of losing our ability to reveal what is distinctive about depressive experience.

## Conclusion

Phenomenological psychopathology has urged us to consider the breakdown of social relations as not simply a result of depression but a core feature of what it is to experience depression. I have sought to enrich this view by not only drawing attention to the isolation and disconnection that is experienced by many depressed people but considering which kinds of social relationship might be impaired in depression. The characteristic feature of we-experiences is that we identify with others as “one of us” and experience various actions and emotions as “ours.” This gives rise to a felt sense of togetherness or connectedness with others as a “we,” of not only sharing a world in a broad sense but of sharing experiences together. Importantly, this feeling of togetherness can arise through the performance of new we-experiences but also where that togetherness becomes a habitual feature of certain relationships. I have suggested that in depression the feeling of being different to and misunderstood by others fixes a person in their first-person singular perspective, thus shutting off their ability to experience things as part of a “we.” Experiencing something as a “we” precisely involves experiencing something that could not be had apart and what is threatened is not only a sense of connectedness with others but an openness to being influenced and entangled with the experiences of others, of having the world unfold to us in new and exciting ways.

Exploring exactly what kinds of social relations might be compromised in depression clearly gives us richer insight into the nature of the disorder, deepening our understanding of the pain and loneliness that depressed individuals often suffer. In turn, this analysis furthers our understanding of the emergence, frustration, and erosion of actual and habitual we-experiences. Adopting this situated approach to depression, though, also helps us to understand how depression might worsen over time, as experiences of disconnection become the norm and one’s very hope of sharing experiences with others seeps away. Situating depression, then, is not only a case of situating depression in the lived experience of depressed individual, but of situating the disorder across *time* as something that dynamically progresses. While phenomenology has a long history of situating and understanding psychopathological disorders in the context of a person’s experience, world, and interactions, more attention needs to be given to the dynamic temporal profile of psychopathological disorders.

## Data availability statement

The original contributions presented in this study are included in the article/supplementary material, further inquiries can be directed to the corresponding author.

## Author contributions

The author confirms being the sole contributor of this work and has approved it for publication.

## Footnotes

1 Having a rich understanding of the lived experiences that people with depression have might also help distinguish between different kinds of depression.

2 For further work on the positive experience of absence, see Roberts (2019) and the classic example from Sartre (2005) of sitting in a café waiting for one’s friend to arrive and their absence being explicitly experienced.

3 For related, but subtly different discussions of experiences of ‘unreality’, also see Broome (2012) discussion of ‘Truman belief syndrome’ where individuals think they are living in a pretend reality and discussions of derealization, e.g., Varga (2012).

4 Stanghellini (2004) also offers a description of schizophrenic patients as lacking a sense of others as people.

5 For simplicity, I will focus on we-experiences that arise between two people. I take it, though, that we can have we-experiences with more than one other person.

6 Note that this is not to suggest that pain can never be shared. While some have argued that sensations cannot be shared, I will remain neutral on this here. It also is not the case that only negative experiences might prevent the emergence of a we-experience. I could be so ecstatically happy about an upcoming date that this happiness also is experienced as something that is not common between us and work to hinder the experience of doing the puzzle together as a we.

7 While I have focused on how a salient affective experience that only one participant might frustrate the emergence of a we-experience, structural differences might also have this impact. For instance, if there is a distinct power imbalance between myself and the person doing the jigsaw, this might hinder my ability to feel myself to be enough ‘like them’ for a we-experience to arise. Thank you to Laura Candiotto for raising this example!

8 One might be concerned about the implication of a sedimented background-feeling of togetherness. It risks sounding like an unconscious emotion, a feeling that we are not aware of. Indeed, Szanto (2018) expresses this very worry. However, I agree with Szanto when he states that it would be a mistake to characterise this as a feeling that we are not aware of. Rather, the habitual feeling of *we*-ness is felt but it does not need to go through an explicit performance of an actual we-experience; it is not that we are not aware of thinking of ourselves as a *we* but that we come to do so in an enduring and background way.

9 For a more in-depth discussion of Walther and habitual we-experiences see: Zahavi and Salice, 2017; Osler, 2020; Wilde, 2021.

10 This might also occur in a less dramatic way. Our habit of feeling and thinking as a *we* could simply fade away if the habit does not come to fruition frequently. This might happen when a couple ‘drifts apart’.
